# Psychosocial Aspects of Deliberate Physical Suicide Attempts by Children and Adolescents

**DOI:** 10.1177/00099228221145106

**Published:** 2023-01-30

**Authors:** Odeta Kinciniene, Rokas Sambaras, Auge Lesinskaite, Virginija Zilinskaite, Sigita Lesinskiene

**Affiliations:** 1 Clinic of Children’s Diseases, Institute of Clinical Medicine, Faculty of Medicine, Vilnius University, Vilnius, Lithuania; 2 Clinic of Psychiatry, Institute of Clinical Medicine, Faculty of Medicine, Vilnius University, Vilnius, Lithuania; 3 Faculty of Medicine, Vilnius University, Vilnius, Lithuania

**Keywords:** physical suicide attempt, adolescent, self-harm, comorbidities, bullying, violence

## Abstract

Suicide is the second most common cause of death among young people in Lithuania. This study aimed to evaluate circumstance, and conditions possibly related to suicide attempt in adolescents. Study was performed at the tertiary Children’s Hospital. Medical documentation of the suicide attempts from January 2011 to April 2018 was analyzed. There were 102 cases included in the study: 83.8% female and 16.2% male patients (average age of 15.02 ± 1.9); 40.6% of patients lived in divorced families, 17.7% in orphanages, 4.2% in foster care; 36.4% suffered from parental alcoholism, and 17.2% experienced suicide in close surroundings; 54.1% experienced bullying or violence (26.0%), and 85.4% showed signs of other types of self-harm, girls more often (*P* < .001). In 52.8% of cases, the attempt was spontaneous, and 34% relapsed. In summary female gender, living in orphanages, and signs of other self-harm were significantly related to suicide attempt.

## Background

Suicides or attempted suicides are problems that are particularly relevant today in Lithuania and all around the world. Based on the data provided by the World Health Organization (WHO), approximately 800,000 people commit suicide annually, and suicide is the second most common cause of death among people aged 15 to 29 years old globally.^1^ The data from the Center for Disease Prevention and Control of the United States of America indicated the same tendency in 2016, that is, suicide is the second most common cause of death among people in the 10- to 24-year age group.^2^ The data published by the Organization for Economic Co-operation and Development (OECD) demonstrate that the number of deaths among people aged 10 to 14 years and 15 to 24 years has been decreasing; however, the number of suicides, as the causes of death, persists.^3^

Suicide or attempted suicide is a very pressing problem in Lithuania. Based on data from the Lithuanian Institute of Hygiene (LIH), approximately 1000 people commit suicide annually in Lithuania, and 26.79% of them belong to the 10- to 19-year age group.^4^ In Lithuania, no studies have been conducted on the psychosocial aspects of deliberate suicide by children and adolescents, despite it being a very current issue. A study carried out in Lithuania from 2010 to 2015 revealed that suicide makes up a quarter of all deaths in the 10- to 19-year age group.^5^

The most common stage of childhood for suicide attempts is adolescence.^6-8^

Numerous studies have established that suicide attempt is more common among girls.^7,9^ Findings in the scientific literature show that girls attempt suicide 3 to 9 times more often as boys; however, boys commit suicide 2 to 4 times more often.^10-12^ The number of boys who die by suicide *de facto* was higher and the ways in which they did it were of an aggressive kind.^12^ The findings in the scientific literature defined the seasonal distribution of pediatric suicide: the greatest number of children attempt suicide is in March, and the predominant suicide season is spring.^13^

Self-harm is pretty common among teenagers in Lithuania as well as in other countries. A study performed in Kaunas in 2009 showed that more than 7.3% of individuals in Lithuania aged under 20 have tried cutting themselves or have performed other forms of self-harm.^14^ According to various studies, approximately 11% to 20% of young people deliberately commit acts of self-harm at least once during their lifetime.^10,15^ The occurrence of such aggressive behavior directed toward oneself, which induces a decision to commit suicide, may be determined by various risk factors. History of at least one previous attempt to commit suicide or self-harm episodes is significant suicide attempt indicators.^16^ The WHO identifies several key reasons for attempting suicides as disturbed relationships with other people, divorce or separation, experiencing abuse or violence, conflicts, use of psychoactive substances (alcohol and drugs), mental health problems, long-term diseases, financial constraints, and trauma or a traumatic event (earthquake, terrorist attack, etc.). Social, psychological, and cultural factors create the environment around the individual, and this may increase the risk of suicide, and the stigma of suicide, which exists in society, discourages many help-seeking people from finding it.^17^ Young people who are admitted to hospitals following suicide attempts often aim to escape their problems, which they cannot face on their own, and they cannot find or do not obtain the required help. These young people strive to show how bad they feel and what kinds of internal conflicts they go through. This is also the way for them to show that they need help. Conflicts with parents or friends and separation from their loved ones are the most difficult things to go through for young people. They are also very sensitive to mockery. To them, suicide seems to be the only possible way out, and after each attempted suicide, the probability that the person will commit suicide increases by 20 times in comparison with people who have not attempted suicide previously.^18^ It is also important to mention that each attempt at suicide or suicide itself has a significant impact on people close to that person: relatives, friends, family members, and other persons faced with suicide.^19^

This study aimed to analyze the circumstances of life that could be factors related to suicide attempts, and signs or indicators of threatening suicide. The hypothesis of the study was that suicide attempt among children and adolescents can be predicted and possibly we have some effective methods to prevent the tragedy.

## Methods

### Study Design

This cross-sectional retrospective study was performed at the Children’s Hospital of Vilnius University Santara Clinics (further, Children’s Hospital) from January 2011 to January 2018. Vilnius is the biggest town and the capital city of Lithuania, and the Children’s Hospital is the biggest tertiary pediatric multidisciplinary clinic in Lithuania too. Case histories were selected according to established, definitive diagnosis codes in the International Classification of Diseases—Australian modification (ICD-10-AM), which is the official method used for health care system statistics of Lithuania. The study was approved by the Regional Ethics Committee of Vilnius University Hospital (protocol No. 18VVR-6304).

### Inclusion and Exclusion Criteria

Inclusion criteria into the study were the patient’s age (under 18), admission to hospital after deliberate suicide physical attempt; health status requiring hospitalization for a living patient; and complete medical documentation. Exclusion criteria were a medical history of unintentional, accidental injuries; deceased patient, lack of indication for hospitalization; and incomplete medical records.

### Outcomes Examined

Due to the aim of the study, demographic data: patients age (continuous variable) and gender (qualitative variable) were collected from medical histories. Life circumstances data (qualitative variables): family structure, for example, divorce story (during the suicide attempt the patients’ lives with only one parent after divorce), living conditions—at home versus foster care/orphanage were collected from parents and foster cares.

### Description of Procedures

Social aspects of life data (qualitative variables): experience of being bullied, violence (experience of physical abuse), parental alcoholism or other addiction, and suicide among close surroundings were collected and evaluated from free-form interview conducted by a psychologist. Health conditions (qualitative variables): concomitant disease diagnosed by specialists, were collected and evaluated from case-histories (only those diseases that patients had at the time of the suicide attempt); signs of self-harm were collected and evaluated from medical examination protocols (cuts, burns signs on body, scars left after self-harm). Circumstances of the attempt data (qualitative variables): spontaneous versus planned decision, first attempt versus relapse were collected and evaluated from “psychosocial assessment protocol for persons surviving a suicide crisis” (the protocol consists of general questions about a person’s age and gender, open questions about current suicidal thoughts/intentions, previous suicidal experiences.) The protocol is conducted by a psychologist or children and adolescent psychiatrist. Method of suicide (divided into more potentially lethal and less potentially lethal) collected and evaluated from medical examination protocols. The season of attempt were collected from medical histories.

### Statistical Approach

Continuous variables were expressed as the mean ± standard deviation, and qualitative data were reported as numbers and percentages. The normality of the variable distribution was tested by the Kolmogorov-Smirnov test. The significance of differences between groups with a normal distribution of parameters was assessed by the Independent Samples *t*-test (compared children mean age by gender groups). Associations between qualitative parameters were tested using the chi-square test or Fisher’s exact test. The difference between the observed monthly suicide attempt distribution and expected frequencies was tested using the chi-square test. The null hypothesis was that the frequency of suicide attempts would be equal for each month. The distribution of family structure was compared with the official data from the Statistical Yearbook of Lithuania showing that 0.5% of persons under 18 live in orphanages. These data were processed using a binomial test (1-sample proportion test). The null hypothesis was that the proportion of children in our study who were living in orphanages is 0.5%. The level of statistical significance was considered to be *P* < .05. Microsoft Excel 2010 and IBM SPSS 20.0 software were used for statistical data analysis.

## Results

According to the medical code classification during the study period, 117 individuals were hospitalized due to deliberate physical suicide attempts at the Children’s Hospital, and 102 cases met the inclusion criteria and were included into the study and evaluated.

### Demographic Data

During the study period, 102 cases of intentional suicide attempts were treated in the Hospital. The incidence of the suicide attempts was distributed from 3 to 20 times per year with the maximal quantities occurring in 2014 and 2017. Six patients were hospitalized due to attempting suicide twice, resulting in a total of 96 persons. There were 86 (84.3%) girls’ and 16 (15.7%) boys’ cases included in the study. The mean age of attempting to commit suicide was 15.02 ± 1.9 years (15.12 ± 1.81 for girls and 14.50 ± 2.36 for boys, *P* = .405). The youngest girl at attempt moment was 9, and the youngest boy was 11 years old.

### Life Circumstances

An analysis of family structure showed that only 37.5% of children who attempted suicide lived with full families at home, and 40.6% of them were from divorced families living at home. By evaluating the life conditions, we revealed that 4.2% lived in foster care and 17.7% lived in orphanages. This is in comparison to official data from the Statistical Yearbook of Lithuania (edition 2019) showing that only 0.5% of persons under 18 live in orphanages. Comparing data from the Statistical Yearbook and our study, we found that living in an orphanage could be a factor related to suicide attempt (*P* < .001).

### Possible Social Factors Associated With Suicide Attempts

**Figure 1.**
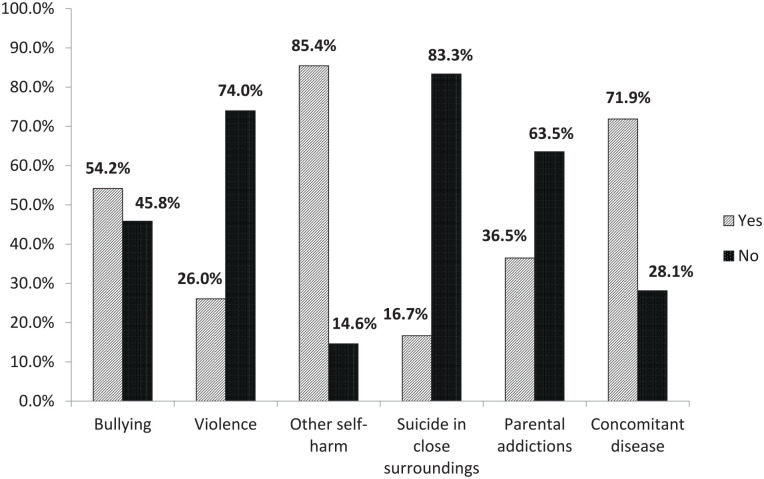
The association of social factors, health conditions related with suicide attempt.

From analyzing the medical history, we found that 52 (54.1%) patients who attempted suicide reported being bullied and 25 (26.0%) had experienced violence. Sixteen children (16.6%) had experience suicide in their close surroundings and 35 (36.5%) reported that their parents have an addiction (alcoholism or other) (Figure 1). Comparison of the Official Statistics of Lithuania and our study data on the impacts of these social factors on suicide attempt was impossible due to the lack of data on the incidences of bullying, violence, and parental addiction in our country.

### Health Conditions

Most of the patients had comorbidities. Concomitant diseases were diagnosed or confirmed by specialists in 69 children (58 [72.5%] girls and 11 (68.8%) boys). The most commonly detected comorbidity was a psychiatric disorder (63; 65.6%) including 25 (26.0%) patients diagnosed with depression. Somatic diseases, such as asthma, diabetes, cerebral palsy, and scoliosis, were diagnosed in 9 (9.4%) patients. Some patients had more than one comorbidity, for example, asthma with depression, scoliosis with a mixed behavioral and emotional disorder, or cerebral palsy with a mixed behavioral and emotional disorder.

There were 82 (85.4%) patients with signs of previous self-harm, such as scars after cutting or burning in various parts of their body. Girls were found to be significantly more susceptible to self-harm than boys: 73 (91.25%) versus 9 (56.25%), *P* < .001, respectively. The frequencies of possible social factors associated with suicide attempts and health conditions in patients after suicide attempts are presented in Figure 1.

### Circumstances of Attempt

The distribution of spontaneous and planned suicide cases was almost equal: 54 (52.9%) were attempted spontaneously, while 48 (47.1%) were planned. Most of the cases 70 (68.6%) had attempted suicide for the first time, but in 32 cases (31.4%), suicide had been attempted repeatedly: 26 were on their second attempt, 5 were on their third, and 1 was on their fourth attempt. Differences between the associations of social factors related to suicide attempt relapse presented in Table 1.

**Table 1.** Association of Possible Social Factors Related to Suicide Attempt Relapse.

	Attempted for the first time	Attempted repetitively	*P*
Suicide case relapse due to gender (n = 102)			
Male	14 (87.5%)	2 (12.5%)	.076
Female	56 (65.2%)	30 (34.8%)	
Suicide in the close surroundings	9 (14.1%)	7 (21.9%)	.333
Parental addiction	22 (34.4%)	13 (40.6%)	.549
Bullying	35 (54.7%)	17 (53.1%)	.885
Violence	16 (25.0%)	9 (28.1%)	.742

There were no significant differences between genders on suicide attempt relapse.

By evaluating the methods selected for suicide attempts between girls and boys, we detected that boys are statistically significantly more likely to choose potentially more lethal methods (*P* =.011). The most common methods used to commit suicide were cutting of the blood vessels and cutting of the forearms (81.4%). Detailed information about selected methods of suicide attempts is presented in Table 2.

**Table 2.** Methods Used for Suicide Attempt and Their Distribution by Gender.

Method of attempt/gender	Girls	Boys	Total
More potentially lethal methods			
Jumping from a height	3 (3.5%)	2 (12.5%)	5 (4.9%)
Hanging	4 (4.7%)	3 (18.7%)	7 (6.8%)
Strangling	4 (4.7%)	1 (6.25%)	5 (4.9%)
Drowning	1 (1.2%)	0	1 (0.98%)
Shooting	0	1 (6.25%)	1 (0.98%)
Total	12 (14%)	7 (43.8%)	19 (18.6%)
Less potentially lethal method			
Cutting forearm	74 (86.0%)	9 (56.2%)	83 (81.4%)
*P*	.011^a^		

a Comparison of girls’ and boys’ choices of more and less potentially lethal methods.

**Figure 2.**
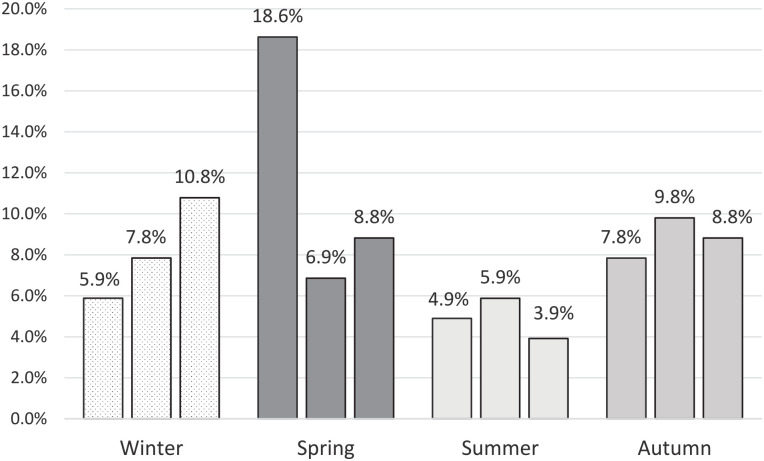
Monthly and seasonal distribution of suicide attempts.

Most suicide attempts were performed in March (18.6%) and February (10.8%), with a total of 34.4% of attempts performed in the early spring period, but there was no significant difference between months (*P* = .688). More detailed information is presented in Figure 2.

## Discussion

According to the literature review as well as our study results, suicidal attempts in the pediatric population are a very real and sensitive problem. In our pilot study, we tried to evaluate possible factors for young people related to suicide attempts. Our evaluated demographic data detected that the average age of children who attempted suicide was 15.02 ± 1.9 years. These data are in accordance with data from many other researchers in Europe and the United States,^6-8,20^ but differ from data collected by African researchers, where the average age of suicide attempt was found to be 17.5 years.^21^ The reason why children start to have suicidal thoughts at this age was explained by a study carried out in Singapore in 2015. The study revealed that children start to experience emotional difficulties during adolescence and learn to control them. However, children do not always manage to control strong emotions due to having weak defense mechanisms, and then, by invoking self-harm or attempting suicide, they attempt to let off accumulated emotional surplus, anxiety, anger, or fears.^22^

During the study, we established that deliberate suicide attempts were more frequent among girls, and the number of boys attempting suicide was almost 4 times less. Despite this, boys used potentially more lethal methods to attempt suicide. These results confirm those obtained in other countries. In a study carried out in 2016 in South Korea, results indicated that the number of boys in the group of children who die by suicide was higher and the ways they did it were of an aggressive kind, i.e., by hanging or jumping from high altitudes. There were more girls in the reference group of children who attempted suicide; however, they did not successfully die by suicide, and the most common method used by girls for die by suicide was self-harm by cutting various body parts.^12^ During our study, it was found that the most common method chosen to attempt suicide was cutting. More aggressive methods, that is, attempts to hang or strangle oneself, jump from a high altitude, drown, or shoot oneself were less frequent, but all were more commonly used by boys. Similar data were reported in a few scientific papers.^23,24^ A study carried out in the United States in 2015 established that the most common methods chosen by adolescents to die by suicide were hanging, self-harm (poisoning or cutting), and shooting. This study also disclosed that the frequency of the methods chosen for die by suicide varies depending on the age of the child, for example, the number of hanging cases proportionally increased with an increase in the age of the child who attempted suicide.^20^ Unfortunately, we could not evaluate this because of the small number of cases with more aggressive methods in our group. The children involved in our study most often tried to commit suicide in March and, less frequently, in February. The peak of hospitalizations due to suicidal attempts was spring, with the rate in autumn being slightly lower. The meta-analysis performed in Greece in 2012 revealed that the seasonal character of suicides indicates serotonin’s responsibility for mood, and its lowest concentration in blood was detected in early spring. In this season, the amount of sunlight increases, resulting in a drastic increase in the blood serotonin level and therefore an increase in the speed of impulse to commit suicide.^25^

By evaluating the living conditions of our study group, we revealed that most of the children who attempted suicide lived with a single parent because their parents were divorced, or, in general, resided outside the family. A considerably lower number of children were living in 2-parent families. In a study carried out in the United States in 2013, young people aged from 13 to 18 years were interviewed to assess the spread of suicidal thoughts and plans. It was established that children who lived in single-parent families had suicidal thoughts more often in comparison to those who lived in 2-parent families.^7^ The research work carried out in the United States in 2010 indicated that the lack of attention from parents and support in the family are key risk factors in determining suicidal thoughts and plans in children.^26^

The results of our study confirm data presented by other researchers showing that life in an orphanage or with foster parents is very traumatic for adolescents and is likely to be a cause of suicidal thoughts and attempts. Based on social security data from the Lithuanian Statistics Department, about 0.8% of all people aged under 18 years in 2016 and 0.5% in 2019 were living in orphanages in Lithuania.^4^ However, our study revealed that suicide attempts were statistically significantly more likely to occur among orphans than among children living in families. This was also confirmed by a meta-analysis performed in England in 2017, where the spread of suicidal ideas, suicide attempts, and suicides committed among adolescents was analyzed. It was determined that the rates of attempted or dying of suicide in children residing in an orphanage were 3 to 4 times higher. There was no difference in the spread of suicidal ideas among children residing in orphanages and children who lived with their parents.^27^ The study of adolescents residing in orphanages or living with foster parents carried out in the state of Colorado in the United States in 2014 disclosed that more than 26.4% of these children had suicide attempts in their medical history, and 4.1% of attempts were lethal. This study also disclosed that a longer period of residence in an orphanage and more frequent changes in place of residence were associated with a greater risk of a child trying to commit suicide.^24^

We noticed a tendency for the number of children living in orphanages in Lithuania to decrease, but the problem is still significant. Unfortunately, we could not find data about the incidence of living in foster care among people aged under 18 years in Lithuania. This practice is very “young” in our country, and we suggest that the occurrence is less than 4.1%, in contrast to our study group.

During our study, possible social factors associated with suicide attempts and risk factors associated with dying of suicide were assessed. Different kinds of humiliation are very common among adolescents living or spending time together as well as during direct contact on internet websites. Our data indicate that more than half of the children who attempted suicide had undergone previous continuous bullying, and about 25% of these children disclosed that they were the victims of abuse or violence. According to official statistics, about 25% of school children are victims of bullying, and 3.5% to 6% of children experience violence at least once. Based on the data, the incidence of bullying and violence is possibly higher among children and adolescents who attempt suicide. Almost one-fifth of the study group had a previous experience of suicide within their close surroundings and more than one-third had experienced parental alcoholism or another kind of addiction. The main reason given by the children as a cause of suicide was a conflict in the family.^28^ A study carried out in the United States in 2001 disclosed that in the prevention of child and adolescent suicides, it is very important to identify suicide risk factors within the domestic environment, such as interpersonal relation issues, family conflict, lack of support, conflict with friends, separations, and suicide cases in domestic surroundings.^29^ Scientific findings show that children with negative emotional and psychological experiences and emotional dissatisfaction are at a significantly higher risk of dying by suicide than children living within a positive emotional environment.^30,31^

Numerous studies have revealed the association of health status with a person’s life quality. During our study, it was disclosed that three-quarters of the study group had comorbidities. In most of the cases, comorbidity disease had a psychiatric origin.

Data from an epidemiological study conducted in Lithuania from 2003 to 2007 showed that 13.1% of children aged 7 to 16 have mental health problems according to ICD-10-AM.^32^ The results of our study revealed that the incidence of comorbidities, especially psychiatric, is several times more common in children who have attempted suicide. In our study, we found that the most common comorbidity disease among the research subjects was depression, and no less than one-third of all children who attempted suicide suffered from depression. The results of the study performed in Lithuania 2002 suggest that 57.4% of the female adolescent suicide attempters and 9.3% of their nonsuicidal peers in the comparison group were diagnosed clinical depression.^33^ The abovementioned disorders, as established in a study carried out in Germany in 2018, are associated with an increased risk of suicide in both adults and children.^34^ A study carried out in the United States in 2015 proposed that 13 to 18 years of age is a critical time frame when depression can develop. There are no comprehensive data on the mechanisms to explain why some children develop depression and some do not; however, it is likely that stressful events, for example, strained interpersonal relations, conflicts with friends, pressure, and genetic predisposition have significant impacts on this. It has also been shown that the reaction of girls to stress originating from interpersonal relations is more intensive, and they are exposed to a higher risk of developing depression.^35^

After assessing other health conditions in our study group members, we found that no less than three-quarters of the children who attempted suicide had self-harmed themselves cutting or burning various body parts. Based on data found in the scientific literature, children who had suicidal thoughts and attempted suicide often self-harmed themselves.^8,24^

The circumstances of attempts were evaluated with attention paid to the speed of making the decision compared with the recurrence of attempt and choice of method for the attempt. The methods of suicide were discussed earlier, but then assessing the data, we found that more than a half of the children had tried to commit suicide spontaneously and the remaining portion had planned their suicide. Research carried out in Denmark in 2016 disclosed that children, in comparison with adults, more often try to commit suicide spontaneously. This finding was explained by the fact that the understanding of time differs in children compared with adults; therefore, when making decisions, only the current difficulties and short-term consequences of their behavior are considered.^36^ Suicide attempt relapse was detected in one-third of our study group. A study carried out in Denmark in 2016 identified previous attempts to commit suicide as a key risk factor in determining potential repeated suicide attempts.^36^ A review of pediatric suicides performed in the United States in 2019 for a 25-year period analyzed child and adolescent suicides and identified that one-quarter of children who die of suicide had previously attempted to commit suicide.^37^

The main limitations of the study are impossibility to evaluate possible factors related to suicide as well related to suicide attempt in adolescents due to lack of those data in the Statistical Yearbook of Lithuania and epidemiological data of the study covering a large party, but not all the country of Lithuania.

## Conclusions

This study is the first to investigate the psychosocial factors of children attempting suicide in Lithuania. Our study observed important circumstances in adolescent suicide attempts. Females were more likely to make an attempt, and males were more likely to choose potentially lethal methods. Other associated factors included a lack of prosperity in family life, experience of bullying and violence, and presence of comorbidities. The critical time for an attempt to occur was found to be early spring. Signs of self-harm could be an indicator before the suicidal attempt.

Considering the relevance of the adolescent suicide issue, intensive and continuous psychiatric/psychological support and care must be given to children who live in an orphanage or are under foster care, have been diagnosed with depression, were or still are bullied, have previously attempted suicide, or repeatedly self-harm themselves by using other methods. Particular attention should be given to these children and adolescents in the late winter and early spring period.

The key message of our study is:

The pediatric suicide sometimes can be predicted and possibly even prevented. Children and adolescents from the unwell families, suspected or detected with different forms of bullying, with signs of self-harm, and particularly after the previous attempt should be given the additional attention from the health and social care service. The data of study can serve as basis for the pediatric suicide prevention program and possibly have value for the cross cultural comparison.

## Author Contributions

OK: Contributed to conceptualization, methodology, writing—original draft, writing—review & editing.

RS: Contributed to formal analysis and investigation.

AL: Contributed to investigation and writing—original draft.

VZ: Contributed to conceptualization, supervision, and methodology.

SL: Contributed to conceptualization and writing—review & editing.
